# Feasibility and acceptability of a cohort study baseline data collection of device-measured physical behaviors and cardiometabolic health in Saudi Arabia: expanding the Prospective Physical Activity, Sitting and Sleep consortium (ProPASS) in the Middle East

**DOI:** 10.1186/s12889-024-18867-2

**Published:** 2024-05-22

**Authors:** Abdulrahman I. Alaqil, Borja del Pozo Cruz, Shaima A. Alothman, Matthew N. Ahmadi, Paolo Caserotti, Hazzaa M. Al-Hazzaa, Andreas Holtermann, Emmanuel Stamatakis, Nidhi Gupta

**Affiliations:** 1 Department of Physical Education, College of Education, King Faisal University, Al-Ahsa, 31982 Saudi Arabia; 2 Center for Active and Healthy Ageing (CAHA), Department of Sports Science and Clinical Biomechanics, University of Southern Denmark, Odense, 5230 Denmark; 3 Department of Musculoskeletal Disorders and Physical Workload, National Research Centre for the Working Environment, Lersø Parkalle 105, Copenhagen, 2100 Denmark; 4 Faculty of Education, Department of Physical Education, University of Cádiz, Cádiz, Spain; 5 Biomedical Research and Innovation Institute of Cádiz (INiBICA) Research Unit, University of Cádiz, Cadiz, Spain; 6 Lifestyle and Health Research Center, Health Sciences Research Center, Princess Nourah Bint Abdulrahman University, Riyadh, 11671 Saudi Arabia; 7 Mackenzie Wearables Research Hub, Charles Perkins Centre, The University of Sydney, Camperdown, NSW Australia; 8 School of Health Sciences, Faculty of Medicine and Health, The University of Sydney, Camperdown, NSW Australia; 9 School of Sports Sciences, University of Jordan, Amman, Jordan

**Keywords:** Feasibility, Epidemiology, Physical activity, Physical behavior, Sedentary behaviors, Accelerometry, Wearables, Saudi adults

## Abstract

**Background:**

Physical behaviors such physical activity, sedentary behavior, and sleep are associated with mortality, but there is a lack of epidemiological data and knowledge using device-measured physical behaviors.

**Purpose:**

To assess the feasibility of baseline data collection using the Prospective Physical Activity, Sitting, and Sleep consortium (ProPASS) protocols in the specific context of Saudi Arabia. ProPASS is a recently developed global platform for collaborative research that aims to harmonize retrospective and prospective data on device-measured behaviors and health. Using ProPASS methods for collecting data to perform such studies in Saudi Arabia will provide standardized data from underrepresented countries.

**Method:**

This study explored the feasibility of baseline data collection in Saudi Arabia between November and December 2022 with a target recruitment of 50 participants aged ≥ 30 years. Established ProPASS methods were used to measure anthropometrics, measure blood pressure, collect blood samples, carry out physical function test, and measure health status and context of physical behaviors using questionnaires. The ActivPal™ device was used to assess physical behaviors and the participants were asked to attend two sessions at (LHRC). The feasibility of the current study was assessed by evaluating recruitment capability, acceptability, suitability of study procedures, and resources and abilities to manage and implement the study. Exit interviews were conducted with all participants.

**Result:**

A total of 75 participants expressed an interest in the study, out of whom 54 initially agreed to participate. Ultimately, 48 participants were recruited in the study (recruitment rate: 64%). The study completion rate was 87.5% of the recruited participants; 95% participants were satisfied with their participation in the study and 90% reported no negative feelings related to participating in the study. One participant reported experiencing moderate skin irritation related to placement of the accelerometer. Additionally, 96% of participants expressed their willingness to participate in the study again.

**Conclusion:**

Based on successful methodology, data collection results, and participants’ acceptability, the ProPASS protocols are feasible to administer in Saudi Arabia. These findings are promising for establishing a prospective cohort in Saudi Arabia.

**Supplementary Information:**

The online version contains supplementary material available at 10.1186/s12889-024-18867-2.

## Background

Global data from 2023 indicate that an estimated 27.5% of adults do not meet physical activity guidelines and have poor physical behaviors (e.g., physical activity, sedentary behavior, and sleep) that are linked with an increased risk of morbidity and mortality [1–4]. Sufficient physical activity and sensible sedentary times are associated with better health outcomes (e.g., cardiovascular health, mental health, and physical function) [1, 2]. Despite this fact, 50–90% of Saudi Arabian adults perform low or insufficient daily physical activity; about 50% spend at least five hours per day sitting [5]. Furthermore, around 33% of the population experiences sleep durations of less than 7 h per night [6]. These trends could be a reason why non-communicable diseases account for 73% of mortality and cardiovascular diseases account for 37% of all deaths among Saudi Arabian adults [7]. However, there have been few studies in Middle Eastern countries, and the evidence that links between physical behaviors and health outcomes is under-represented in Saudi Arabia [1].

Furthermore, within Saudi Arabia, the few studies exploring this connection often rely on self-reported physical behaviors that often do not provide the most accurate picture [5, 8–11]. This lack of data necessitates studies that incorporate measurements from devices that directly track these behaviors among Saudi Arabian adults, which aligns with recent guidance from the World Health Organization (WHO) on the necessity of incorporating device-measured physical behaviors into future studies to explore their relationships with various health aspects [1, 12]. By employing such a method, we can gain more precise insights into the dose-response relationships between different physical behaviors and various health outcomes among Saudi Arabian adults.

The Prospective Physical Activity, Sitting, and Sleep Consortium (ProPASS) is an initiative that aims to explore how thigh-based accelerometry measurement of physical behaviors influences a wide range of health outcomes. This initiative operates on a global scale and aims to harmonize data from both retrospective and future studies [13]. To fulfill the aim, ProPASS is developing methods for collecting prospective data and processing, harmonizing, and pooling data from previous and future studies [14]. To date, the methods of the ProPASS consortium have been used to harmonize data from large-scale epidemiological studies, such as the 1970 British Birth Cohort, the Australian Longitudinal Study on Women’s Health [15], and Norway’s Trøndelag Health Study (HUNT) [16, 17]. As such, this study seeks to determine if the ProPASS methodologies will be effective in the context of data collection within Saudi Arabia. This will be beneficial because it will help to standardize the measurement of physical behaviors, enhance harmonization across studies, and create more a representative and valid understanding of the associations between physical behaviors and health globally, including under-represented countries such as Saudi Arabia.

This paper describes the feasibility of baseline ProPASS data collection in Saudi Arabia with prospectively harmonized data with the main resource. This feasibility study of baseline data collection will serve as a framework for a future cohort study that will investigate the associations between device-measured physical behavior (e.g., physical activity, sedentary behavior, and sleep) and cardiometabolic health in Saudi adults.

## Method

The study was approved by the Institutional Review Board at Princess Nourah Bint Abdul Rahman University, Riyadh, Saudi Arabia (IRB 22–0146), and was carried out in accordance with the principles of the Declaration of Helsinki.

### Study design and procedures

**Fig. 1:**
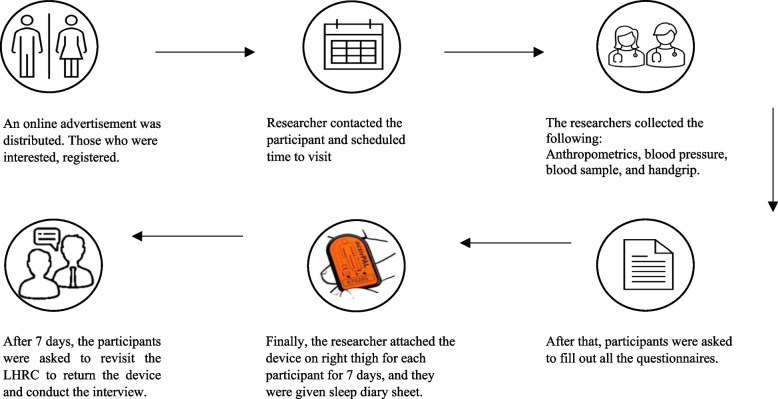
Demonstration and summary of the study procedure

Participants were informed about the study’s aims and asked to read and sign the consent form before any measurements were taken. After agreeing to participate, they were asked to attend two sessions at the Lifestyle and Health Research Center (LHRC) at the Health Sciences Research Center of Princess Nourah Bint Abdulrahman University. During the first visit, each participant’s anthropometric measurements (e.g., height, weight, waist circumference), blood pressure and heart rate, blood samples, and handgrip strength were measured. Next, the participants completed questionnaires on demographic information, dietary habits, self-rated health, self-reported smoking status, and the Global Physical Activity, Sedentary Behaviors, and Sleep behavior questionnaires. At the end of the first visit, the researcher attached the ActivPAL™ accelerometer device to their thigh which they were asked to wear for seven consecutive days. Participants were also provided with a diary to record their waking and sleeping hours [18]. On the 8th day of study, the participants were asked to attend the LHRC for session two where they returned the device and were interviewed (see Fig. 1).

### Participants and eligibility

The study aimed to recruit a total of 50 Saudi adults aged ≥ 30 years, which is generally considered a common sample size for feasibility studies [19, 20]. The eligibility criteria were: (1) Saudi nationals (2), resident in Riyadh, and (3) aged ≥ 30 years old. The exclusion criteria were: (1) having a current medical condition that forces them to be chair-bound or bedridden for more than half of their waking hours (2), being allergic to plasters or adhesives (3), being allergic to low-density polyethylene (4), having a skin condition that would prevent them from wearing the monitor, and (5) those who may need to pass through a metal detector/security checkpoint during the duration of the study. The study’s aims, protocol, and procedures were clearly described to all participants before any measurements were taken.

### Recruitment

Participant recruitment was carried out over the month of November 2022. Participants were recruited from different locations across Riyadh, Saudi Arabia, by using electronic flyers on social media (e.g., Twitter, WhatsApp) that provided information about the study and the researcher’s contact details. Prospective participants who were interested in joining the study were asked to provide their contact information via a link to Google Forms featured in the study description. The participants who initially expressed interest but later decided not to join were invited to share their reasons for non-participation through a physical or telephonic meeting.

### Measurements based on ProPASS methodology

The current study employed the ProPASS method and protocol for new cohort studies that seek to join ProPASS prospectively [14, 21]. All measurements were taken by researchers that were well-trained in the ProPASS protocol and methods. Blood pressure and hand grip strength measurements were taken three times, and the mean average was then calculated; all other measurements were taken only once.

#### Anthropometric measurements

Height (to the nearest 0.1 cm) and weight (to the nearest 0.1 kg) were measured with a stadiometer (SECA 284; Seca, Hamburg, Germany), and scale (SECA 284; Seca, Hamburg, Germany), respectively. Waist circumference (to the nearest 0.1 cm) was measured midway between the lower rib margin and the iliac crest at the end of a gentle expiration [22]. Body mass index (BMI) was calculated using the standard calculation (height in meters squared/body weight in kilograms).

#### Blood pressure and heart rate

Blood pressure was taken after resting for five minutes in a sitting position. Blood pressure was taken three times with one minute between measurements and the average reading was recorded [23]. Blood pressure and heart rate were measured using a Welch Allyn Connex 7300 Spot Vital Signs Monitor, which provides a high degree of accuracy [24]. Mean arterial pressure (MAP) was then calculated (MAP = 1/3 * SBP + 2/3 * DBP in mm Hg) using the average of both the SBP and DBP values [25].

#### Blood samples

Non-fasting finger-prick (capillary) blood samples (40 µL) were collected for analysis after warming the finger for five minutes. A drop of blood was taken directly from the heated finger to be analysed for blood glucose, triglycerides, total cholesterol, high-density lipoprotein cholesterol, and low-density lipoprotein cholesterol. A previously validated CardioChek PA analyser (CardioChek PA Blood Analyser, UK) was used to analyse the blood samples [26, 27].

#### Medication use

Participants’ medication use was evaluated by the question: *Do you currently use any prescription medicines*? If the answer was yes, the participants were asked which medications they use, such as medication for high blood pressure, high cholesterol, asthma, COPD, anxiety, depression, thyroid problems, allergies. They were also asked whether the medication was in the form of tablets, or nasal sprays, whether the medication was anti-inflammatory, chemotherapeutic, urological, birth control, or neurological, and the age at which the participants had begun using the medication.

#### Familial disease history

Familial disease history was assessed by the question: *Do your parents, siblings or children have, or have they ever had, some of the following diseases before the age of 60*? The responses included asthma, hay fever/nasal allergies, chronic bronchitis, emphysema or COPD, anxiety or depression, myocardial infarction (heart attack), diabetes, stroke or brain hemorrhage, and cancer. The responses were *yes, no*, and *I don’t know*.

#### Chronic health status

Participants’ chronic disease status and/or long-term health issues were assessed by the question: *Have you had, or do you have any of the following diseases?* The responses included angina, myocardial infarction (heart attack), heart failure, peripheral vascular disease, atrial fibrillation, stroke/brain hemorrhage, thrombosis, pulmonary embolism, asthma, COPD or emphysema, diabetes, hypothyroidism (low metabolism), hyperthyroidism (high metabolism), cancer, migraine, psoriasis, kidney disease, arthritis (rheumatoid arthritis), Bechterew’s disease, gout, mental health problems, osteoporosis, sleep apnea, arthrosis, nerve disease, hearing/ear disease, eye disease, and infection. Those who replied yes were asked a follow-up question: *How old were you when you had it for the first time?*

#### Mobility limitations

The questionnaire was based on three questions on performance-based measures of mobility, which had already been translated and culturally adapted into Arabic [28]. These three questions are valid and reliable tools to identify the early indications of disability and can be used as indicators to identify those at high risk of future disability [29]. Self-reported mobility was assessed via the following questions: (1) *Do you have difficulty in walking 2.0 km?* (2) *Do you have difficulty in walking 0.5 km*? and (3) *Do you have difficulty in walking up one flight of stairs?* The five response options were: (1) *able to manage without difficulty* (2), *able to manage with some difficulty* (3), *able to manage with a great deal of difficulty* (4), *able to manage only with the help of another person, and* (5) *unable to manage even with help.*

#### Dietary habits

The dietary habits questionnaire was translated and culturally adapted into Arabic [28]. The questionnaire assessed the dietary habits of the participants was adapted from the Survey of Health, Aging, and Retirement in Europe (SHARE), which has been demonstrated to be a valid and reliable tool for assessing diet [30]. The questionnaire focused on the consumption of dairy products, legumes, eggs, meat, fruit and vegetables.

#### Self-rated health

A set of valid and reliable questions adapted from Idler et al.’s (1997) questionnaire was used to assess participants’ self-rated health by asking them to rate their health status using the following questions: (1) *In general, would you say your health is…: Excellent; Very good; Good; Fair; Poor;* (2) *Compared to one year ago, how would you rate your health in general now?: Much better now than one year ago; Somewhat better now than one year ago; About the same; Somewhat worse now than one year ago; Much worse now than one year ago* [31, 32].

#### Smoking habits

Self-report questions on smoking behavior were adapted from the UK Biobank questionnaire and were used to assess participants’ present and past smoking habits including at what age they began smoking. the number of cigarettes smoked per day, the type of tobacco used, the duration of smoking, and, among former smokers, the age when smoking ceased [33].

#### Physical behaviours

Physical behaviors such as physical activity, sedentary behavior, and sleep were measured by using (1) self-reported and (2) device-based measures:

#### Self-report measures

Physical activity was measured on a self-report basis via the Global Physical Activity Questionnaire (GPAQ) which was translated into Arabic and previously validated [34]. In addition, the Sedentary Behavior Questionnaire (SBQ), which had already been translated into Arabic [28], was used to subjectively assess participants’ sedentary behavior time [35]. Lastly, the Pittsburgh Sleep Quality Index was used to assess sleep quality and sleep disturbances over a one-month period [36].

#### Device-based measures

Physical behaviors were measured by wearing a thigh-worn accelerometer device (an ActivPAL™ Micro4, PAL technologies, Glasgow, Scotland) that participants wore continuously for 24 h for seven full days [37]. The Activpal™ device was sealed with a nitrile sleeve and attached with a medical waterproof 3 M Tegaderm transparent dressing on the front of the right mid-thigh on the muscle belly by a well-trained member of researcher team. The ActivPAL™ monitor is a valid and reliable measure of time spent walking [38], sitting, and standing time in healthy adults [39]. In addition, the participants were asked to fill in a recording sheet that included a sleep diary (times that the participant went to and got out of bed), as well as, the dates and times when the accelerometer fell off or was removed.

#### Physical function

Physical function was objectively measured using a digital hand-grip strength dynamometer (Takei Hand Grip Dynamometer 5401-C, Japan) via three successive hand-grip assessments for each hand (left and right); the mean value for each hand was then recorded. The instrument can measure hand-grip values from 5 to 100 kg; the minimum unit of measurement is 0.1 kg. The tool is a good health outcomes predictor [40, 41].

### Data collection evaluation of feasibility

Overall, the study evaluated feasibility in two main stages where feedback from the first six participants was used to resolve any unforeseen issues in the protocol implementation on the remaining participants. Any changes to the procedure were documented.

The current study evaluated the feasibility of Saudi adults’ participation based on the following constructs: (1) recruitment capability (2), acceptability and suitability of study procedures, and (3) resources and ability to manage and implement the study. Table 1 outlines the feasibility constructs, measures, outcome definitions, and methods employed. In evaluating feasibility, the current study followed the recommendations for a feasibility study as reported by Orsmond and Cohn, 2015 [42].

**Table 1:** Evaluation domains of the current feasibility study

Feasibility construct	What are you measuring	Definition	Method
**Evaluation of recruitment** **capability**	Recruitment rate	• % of participants who register • % of participants who scheduled an appointment • % of participants who show up • % of participants who completed	Tracking the registration
	Recruitment barriers and facilitators	• No more interested in participating in the study.	By personal contact during recruitment
**Acceptability, Suitability, of Study procedures**	Adherence rate	• Number of days with accelerometer recording • % of days with diary registrations • Number of valid days of accelerometry data • % of completed questionnaires	Processing sensor data and questionnaire entries
	Time burden	• The participants estimate of time uses on: Beginning measurement on day 1 all measurements (questionnaires + attach the sensor)	Tracking the time during the first day
	Completion rate	• % of registered participants completing all days of accelerometer measurement, diary registration, and all questionnaires	Tracking the completion
**Resources and ability to manage and implement the study**	Skin irritation	• Number of participants reporting skin irritation	Personal contact during the study
	Equipment availability	• “Is equipment available when needed?”	Tracking time before data collation
	Training requirements	• The amount of time took to train the researchers	
	Accelerometer lost	• The amount of data lost due to mechanical problems, failures, or not returned	Tracking during and after data collation

Overall, the study collected data on the feasibility constructs via tracking the registration, equipment availability, and time spent on various tasks performed (for example training researchers, performing various tasks like attaching the sensor) and completion rate (such as tracking diary entries, questionnaire entries and number of days with accelerometer data), via personal contacts (for information on barriers and facilitators of participation), via processing sensor data, and via interviews after the measurement (for example obtaining information on potential issues during measurement and willingness to participate).

### Participant interviews after measurement

After the completion of the study, face-to-face semi-structured interviews were conducted with all participants who had completed the 7-day study period. The aim of these interviews was to collect comprehensive feedback regarding participants’ experiences with the study protocol, with the goal of capturing additional insights that was not captured by other feasibility measures. Some examples of such measures were motivations for joining the study, their expectations prior to participation, and their levels of satisfaction with the study procedures. A detailed interview guide is described in Appendix A [28, 43, 44].

### Statistical analysis

**Fig. 2:**
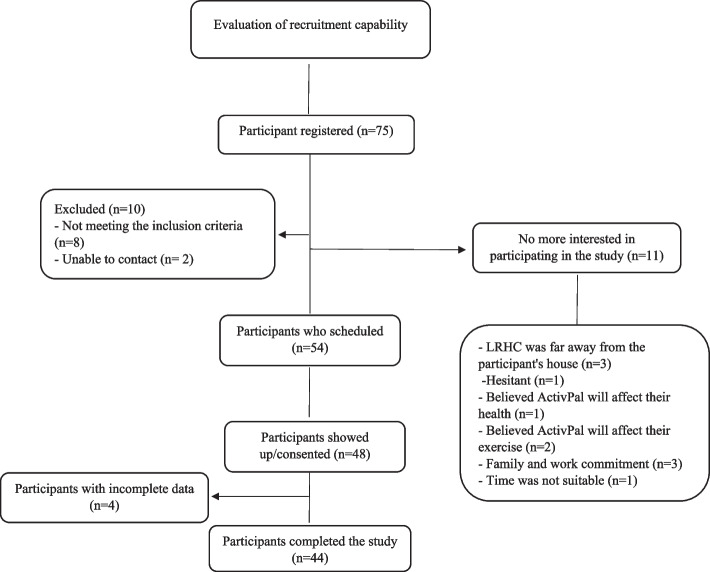
Recruitment and study participant’s diagram

Descriptive analysis summarized participants’ demographics, anthropometric measurements, health status, clinical measurements, physical behaviors characteristics, and interview questions responses. The continuous variables were characterized using mean ± standard deviations (SD), while categorical variables were presented using frequencies accompanied by percentages (%). The recruitment rate was calculated by the number of participants who participated and signed the consent form / total number of participants who registered in the study (see Fig. 2). Additional analyses were performed to compare participants who reported burden with those who reported no burden of participation (see supplementary materials). T-tests and Chi-square tests were employed for this comparison. IBM’s Statistical Package for the Social Sciences (SPSS) (version 27 SPSS, Inc. Chicago, Illinois) was used to conduct the qualitative analysis. The raw data of ActivPAL were analyzed by using the ActiPASS software (ActiPASS © 2021 - Uppsala University, Sweden).

## Results

### Recruitment

A total of 75 participants initially volunteered to participate. Ten participants were excluded from the study as they did not meet the inclusion criteria (*n* = 8) or could not be contacted (*n* = 2). In addition, 11 participants withdrew their interest in participating for various reasons: (1) excessive distance between the location of the study (LRHC) and their residence (*n* = 3) (2), hesitant about joining the study (*n* = 1) (3), believed that the ActivPAL™ device would interfere with his/her health (*n* = 1) (4), believed that the ActivPAL™ device would interfere with their regular exercise routine (*n* = 2) (5), had family and work commitments (*n* = 3), and (6) claimed that the timing was unsuitable (*n* = 1). Out of a total of 54 participants who had agreed to participate in the study, 48 participants from Riyadh, Saudi Arabia, attended and completed the consent form. However, four of those participants provided incomplete data (i.e., they completed the questionnaires only and did not wear an ActivPAL™ device). Therefore, a total of 44 participants out of 75 potential participants (59%) successfully completed the study (wore an ActivPAL™ device and completed all questionnaires). See Fig. 2 for the study’s recruitment flow.

### Participants

Of the 48 participants, nearly half were female (47.9%). On average, the participants were 37 ± 7.3 years old, had a BMI of 28.3 ± 5.6, and a waist circumference of 86.9 ± 16.4 cm. Most participants were married, had college degrees, were employed as office workers and professionals, had never smoked, and did not use any medication (see Table 2). A total of 87.5% of participants had a family history of disease; 85.4%, 95.8%, and 89.6%, reported having no difficulty walking 2 km, 500 m, and up one flight of stairs, respectively. Approximately 48% of participants rated their health as *very good*, while 39.6% reported their health *as about the same compared to one year ago*. In terms of dietary habits, nearly half the participants reported consuming dairy products every day, 25% consumed legumes and eggs 3 to 6 times a week, 56.3% consumed meat every day, and 45.8% consumed fruits and vegeTables 3, 4, 5 and 6 times a week.

**Table 2:** Overall outcomes of the sample population frequency (n) and percentage (%)

**Variables**		**Mean ** **± SD/ Frequency (%)** ^*^					
**Age (years)**		38±7.31					
**Height (cm)**		166.48±10.10					
**Weight (kg)**		78.71±18.84					
**Body Mass index (kg·m** ^**-2**^ **)**		28.26±5.61					
**Waist circumference (cm)**		86.87±16.36					
**Sex**	Female	23 (47.9)					
	Male	25 (52.1)					
**Marital Status**	Single	16 (33.3)					
	Married	30 (62.5)					
	Divorced	2 (4.2)					
**Geographical Location**	North	26 (54.2)					
	South	3 (6.3)					
	East	10 (20.8)					
	West	9 (18.8)					
**Education**	High School	4 (8.3)					
	College degree	28 (58.3)					
	Post-graduate degree	16 (33.3)					
**Monthly Income (Saudi Riyal)**	10000 or less	11 (22.9)					
	10001 to 20000	26 (54.2)					
	20001 to 30000	3 (6.3)					
	30001 and more	5 (10.4)					
**Employment status**	Employed	44 (91.7)					
	Unemployed	1 (2.1)					
	Homemaker	1 (2.1)					
	Unpaid voluntary work	1 (2.1)					
	Retired	1 (2.1)					
**Work type**	Desk Job	24 (50)					
	Physical	6 (12.5)					
	Mixed	17 (35.4)					
**Occupational group**	Manager	9 (18.8)					
	Professional	17 (35.4)					
	Clerical support workers	12 (25)					
	Service and sales workers	1 (2.1)					
	Craft and related trades workers	2 (4.2)					
	Armed forces occupations	2 (4.2)					
**Smoking Habits**	Never smoked	34 (70.8)					
	Used to smoke, but not anymore	4 (8.3)					
	Only smoke occasionally	5 (10.4)					
	Currently smoke on most or all days	5 (10.4)					
**Number of Medication **	No	27 (56.3)					
	One	11 (22.9)					
	More than one	10 (20.9)					
**Number of Chronic diseases**	No	30 (62.5)					
	One	9 (18.8)					
	More than one	9 (18.8)					
**Familial disease history**	Has family disease history	42 (87.5)					
	No family disease history	6 (12.5)					
**Mobility limitations**		2-Km		500m		1-flight of stairs	
	No difficulty	41 (85.4)		46 (95.8)		43 (89.6)	
	Difficulty	7 (14.6)		2 (4.2)		5 (10.4)	
**Health Rated**		**Excellent**	**Very good**		**Good**	**Fair**	
	In general, would you say your health	14 (29.2)	23 (47.9)		8 (16.7)	3 (6.3)	
		**Much better now**	**Somewhat better now**		**About the same**	**Somewhat worse now**	**Much worse now**
	Compared to one year ago	11 (22.9)	6 (12.5)		19 (39.6)	9 (18.8)	3 (6.3)
**Dietary Habits**		**Everyday**	**3-6 times a week**		**Twice a week**	**Once a week**	**Less than once a week**
	Dairy Product	23(47.9)	11(22.9)		8(16.7)	1(2.1)	5(10.4)
	Legumes and Eggs	10(20.8)	12(25)		11(22.9)	9(18.8)	6(12.5)
	Meat	27(56.3)	15(31.3)		4(8.3)	0(0)	2(4.2)
	Fruits and Vegetable	12(25)	22(45.8)		6(12.5)	2(4.2)	6(12.5)

Table 3 presents the primary variables of the study: including average systolic, diastolic, and mean arterial pressure values of 121.13 ± 11.81 mmHg, 79.26 ± 8.92 mmHg, and 93.15 ± 9.20 mmHg, respectively. The mean resting heart rate was 74.3 ± 12.66. Furthermore, the non-fasting blood profile of the sample was analyzed and showed the following values: total cholesterol: 177.89 ± 33.79 mg/dL; HDL-cholesterol: 50.96 ± 13.02 mg/dL; triglycerides: 123.94 ± 68.92 mg/dL; LDL-cholesterol: 103 ± 29.89 mg/dL; TC/HDL-cholesterol ratio: 3.71 ± 1.11; LDL/HDL-cholesterol ratio: 2.19 ± 0.81; non-HDL-cholesterol: 127.06 ± 33.51 mg/dL; non-fasting glucose: 102.98 ± 35.36 mg/dL. Table 3 provides an overview of the participants’ physical activity related behaviors.

**Table 3:** Main study variables of the sample population Mean ± SD/ Frequency (%)

Variable	*n*	Mean ± SD	Min-Max
**Blood Pressure and Heart Rate**			
Systolic blood pressure (mmHg)	47	121.13 ± 11.81	99–151
Diastolic blood pressure (mmHg)	47	79.26 ± 8.92	65–119
Mean Arterial Pressure (mmHg)	47	93.15 ± 9.20	76–129
Heart Rate (BPM)	47	74.30 ± 12.66	51–109
**Blood Sample**			
Total cholesterol (mg/dL)	47	177.89 ± 33.79	112–259
HDL-cholesterol (mg/dL)	47	50.96 ± 13.02	27–82
Triglycerides (mg/dL)	47	123.94 ± 68.92	51–337
LDL-cholesterol (mg/dL)	46	103 ± 29.89	41–180
TC/HDL-cholesterol Ratio	47	3.71 ± 1.11	1.7–6.7
LDL/HDL-cholesterol Ratio	46	2.19 ± 0.81	0.6–4.1
Non-HDL-cholesterol	47	127.06 ± 33.51	51–206
Non-fasting Glucose (mg/dL)	47	102.98 ± 35.36	50–306
**Physical activity** ^**a**^			
Vigorous Intensity (METs.min/week)	48	533.5 ± 1157.88	0-7680
Moderate Intensity (METs.min/week)	48	560.9 ± 615.18	0-2880
**Sedentary behavior** ^**b**^			
Weekdays (hours/day)	48	67.1 ± 35.96	15–195
Weekend (hours/day)	48	18.5 ± 10.50	4–63
**Sleep** ^**c**^			
Sleep Duration (hours)	48	7.41 ± 1.64	5–12
Sleep Quality	48	5.27 ± 2.45	1–11
Physical behaviors ****			
Moderate or vigorous physical activity (min/day)	41	58.35 ± 18.91	
Low-physical activity (min/day)	41	65.04 ± 24.29	
Valid Duration (min/day)	41	1272.65 ± 53.99	
Time of lying (min/day)	41	157.77 ± 68.65	
Time of sitting (min/day)	41	478.29 ± 114.55	
Time of standing (min/day)	41	119.39 ± 50.62	
The time of move (min/day)	41	48.60 ± 20.90	
Walking Slow (min/day)	41	16.03 ± 7.58	
Walking Fast (min/day)	41	51.50 ± 18.12	
Time of stair-walking (min/day)	41	3.33 ± 1.77	
Time of running (min/day)	41	2.12 ± 6.84	
Time of cycling (min/day)	41	1.29 ± 3.90	
**Physical Function**			
Handgrip Right (Kg)	44	31.25 ± 10.59	16-53.9
Handgrip Left (Kg)	42	29.89 ± 10.43	14.2–47.5

^a^Global Physical Activity Questionnaire

^b^Sedentary Behavior Questionnaire

^c^ActivPAl™

### Feasibility evaluation

The following results highlight the approaches taken by the current study to assess the feasibility of baseline data collection using ProPASS methodology specifically in the context of Saudi Arabia.

The evaluation of the feasibility of the study protocol was conducted in two stages, initially involving six participants, whose feedback was used to refine and improve the protocol implementation for the remaining participants. Of the six selected participants, three were female. In the pre-evaluation, only two minor issues were encountered; (1) accessing the lab outside of working hours (16:00–22:00) as most participants were unable to attend during the day (07:00–16:00) due to work commitments. This issue was resolved in all subsequent data collection points by receiving approval for extended lab hours; (2) obtaining the required number of ActivPAL™ devices from the technical coordinator due to miscommunication and high demand by other researchers. To prevent further issues, the author obtained 30 devices in advance for the feasibility evaluation.

#### Recruitment capability

The recruitment rate was used to measure the feasibility of recruitment methodology to collect baseline ProPASS data; the results showed that 64% (*n* = 48) of participants signed the consent form and attended the LRHC lab (see Fig. 2). After screening the eligibility criteria, out of a total of 75 participants, 65 met the study criteria, and 11 were excluded from participating due to the reasons as detailed in Fig. 2. As Fig. 2 illustrates, although 54 participants scheduled an appointment for the study, only 48 (64%) attended and signed the consent form. In the final stage of the recruitment process, around 59% (*n* = 44) of participants completed all the required measurements for the study.

#### Acceptability and suitability of study procedures

The adherence rate (i.e., the extent to which participants adhered to the outlined procedures in terms of the number of days with valid accelerometry data) was 5.7 days. Furthermore, participants provided sleep diary entries for 85.4% of days. All questionnaires were completed with a 100% response rate.

To assess the study’s time demands on participants, the length of time participants needed to complete all measurements was mean time of 25 min (23 min to complete the questionnaires and two minutes to attach the sensor). Additionally, the completion rates for the registered participants who completed all the required measurements (i.e., accelerometer measurement, diary registration, and questionnaires) was 91.6%. (See Table 4).

**Table 4:** Feasibility outcome of acceptability, suitability, of study procedures

Feasibility domain	Measured by	Results
Adherence rate	Number of days with accelerometer recording	5.8 days
	% of days with diary registrations	85.4%
	Number of valid days of accelerometry data	5.7 days
	% of completed questionnaires	100%
Time burden	The participants estimate of time used on (completing questionnaires + attaching the sensor)	25 min
Completion rate	% of registered participants completing all days of accelerometer measurement, diary registration, and all questionnaires	91.6%

#### Resources and ability

The final feasibility outcomes (i.e., having the required resources and ability to manage and implement the study) are presented in Table 5. This objective was assessed based on four domains: skin irritation, equipment availability, training requirements, and accelerometer loss (see Table 5). The first domain revealed that three participants experienced skin irritation during the study; of these, two participants had mild symptoms, such as itchiness and discomfort that lasted for the first three days but did not lead to their withdrawal from the study. However, one participant reported moderate irritation resulting in red skin which required them to withdraw from the study. The second domain, equipment availability, indicated that all the necessary equipment was available 100% of the time. The third domain was training requirements, and the researchers required four hours of training on how to use it correctly. Finally, in the accelerometer loss domain, the study recorded four failed devices out of 30 that did not generate data for seven days.

**Table 5:** Feasibility outcome of resources and ability to manage and implement the study

Feasibility domain	Measured by		Results
Skin irritation	Number of participants reporting skin irritation	Mild	2
		Moderate	1
Equipment availability	“Is equipment available when needed?”		100%
Training requirements	The amount of time took to train the researchers		4 h
Accelerometer lost	The amount of data lost due to mechanical problems, failures, or not returned		4 failures

### Participant interview after measurement

**Fig. 3:**
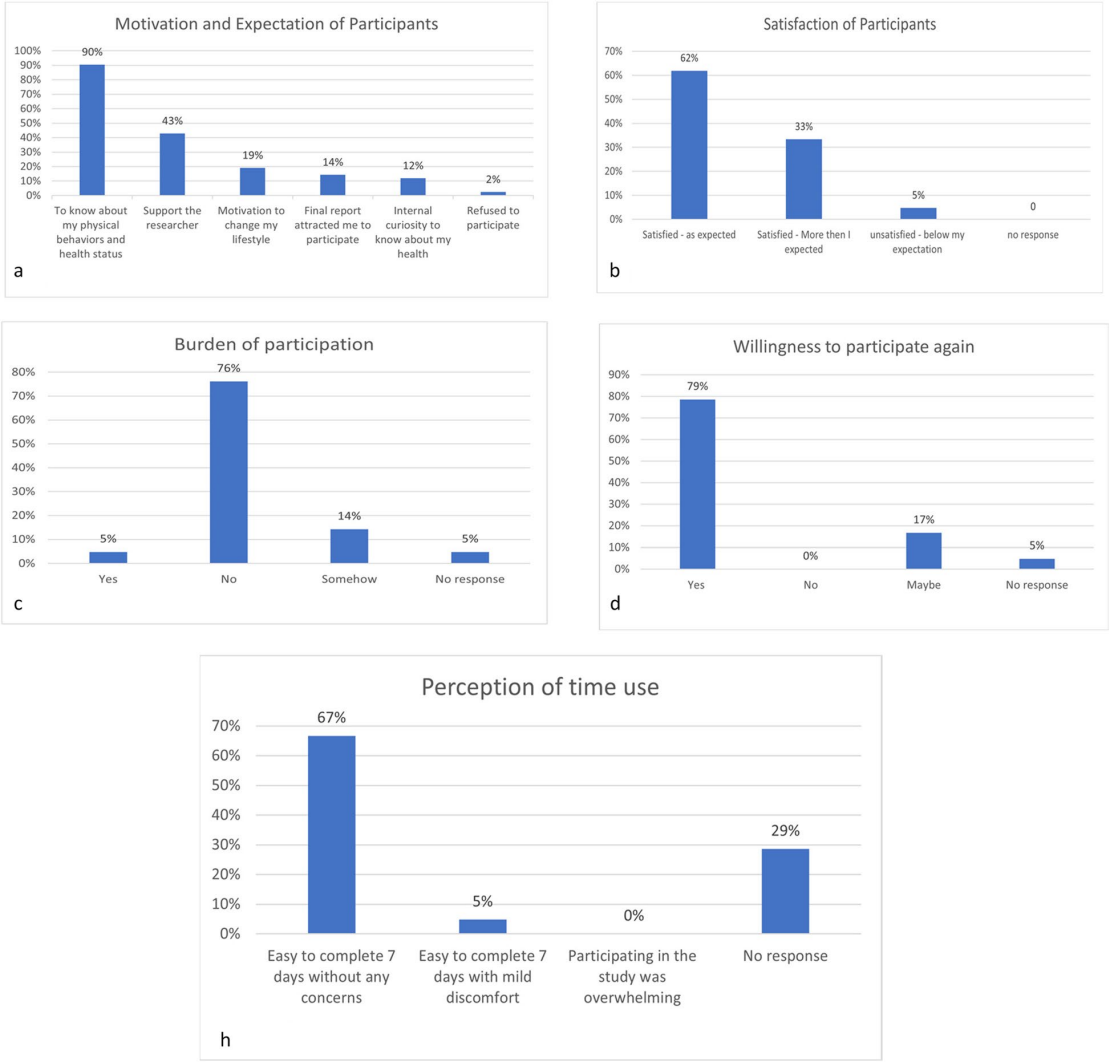
Interview outcomes of participant’s experience with the study protocol

After completing the study, all participants were interviewed around five primary themes: (1) *motivation and expectations of participation* (2), *participant satisfaction* (3), *the burden of participation* (4), *willingness to participate again*, and (5) *perception of time usage* (see Fig. 3).

To determine the participants’ motivations for and expectations about joining the study, they were asked: *What made you want to join this study?* The results showed that 90% of participants were interested in learning about their physical behaviors and health status; 43% participated in supporting the researcher, and 14% reported that the final report attracted them to participate (see Fig. 3a and the example of final report in supplementary material). Participant satisfaction was assessed via two questions: (1) *What was your overall experience of participating in the study?* and (2) *Was it as you expected?* The findings indicated that 62% of participants were satisfied that the study was as expected, 33% were more satisfied than expected, and 5% were unsatisfied and found the study below their expectations (see Fig. 3b).

Regarding the overall burden of participation, 76% of participants reported that it *was no burden*, 5% reported that it *was a burden*, and 14% believed it was somewhat burdensome (see Fig. 3c). Additionally, 79% of participants expressed their willingness to participate again in the future (see Fig. 3d). Finally, regarding time usage, 67% of participants found it easy to complete the seven-day study without any concerns (see Fig. 3h).

## Discussion

The feasibility of the baseline ProPASS data collection methodology was evaluated among Saudi adults who participated in this study. The findings revealed that the methodology was both feasible and acceptable, paving the way for large-scale prospective cohort research in Saudi Arabia. This research marks the first attempt to establish a prospective cohort study in Saudi Arabia using established ProPASS methods [13, 15] and protocols. Conducting such a cohort study in Saudi Arabia is crucial due to the country’s high prevalence of non-communicable diseases that are mostly due to poor physical behaviors (e.g., lack of physical activity, sedentary behavior, and sleep) [7], due to recent enormous economic growth accompanied by technological transformations and urbanization [11].

The first aspect of feasibility evaluated of the baseline ProPASS data collection methodology was the capability to recruit participants. The findings indicated that the recruitment rate was 64% which is similar to prior studies [46, 47]. One study indicated that a recruitment rate of at least between 20 and 40% is required to be deemed feasible [48]. Thus, the recruitment rate in the current study seems acceptable for creating a future cohort using ProPASS methods in Saudi Arabia. Additionally, in the current study, the refusal rate was only 15% which is significantly lower than in previous studies [45, 49] where refusal rates ranged from 50 to 66%. One reason for the low refusal rate in the current study is that the recruitment was material specifically designed to motivate Saudi participants to join the study by indicating that the study would provide data and insight into their current state of health. For example, the results of the semi-structured interviews illustrated that 90% of participants joined the study because they wanted to know about their physical behaviors and health status (see Fig. 3). This result also indicates that our recruitment material might be suitable for ensuring high participation in the future cohort study.

The second aspect of feasibility for the baseline ProPASS data collection methodology that was evaluated in this study was the acceptability and suitability of the study procedures. Previous studies have shown that in order to obtain reliable estimates of adults’ habitual physical activity, it is necessary to record accelerometer data for 3–5 days [50, 51] to gather valid data to perform analysis and provide information about the habitual physical behaviors. A recent study indicated that distributing accelerometers in person was associated with a high proposition of participants consenting to wear an accelerometer and meeting minimum wear criteria [21]. Our study was able to collect an average six days of valid data which was sufficient to obtain representative descriptions of the participants’ physical behaviors [52]. There were high general adherence rates for participant diary entries, questionnaires completion, and adherence to the study protocol, indicating that the ProPASS methods could be feasibly implemented with a larger study population. The study also assessed the time commitment necessary to complete the questionnaires and attach the ActivPAL™ devices to participants’ thighs. Completing the questionnaires took approximately 23 min (SD = 8). Prior studies have indicated that shorter questionnaires (e.g., 20 min) yield a higher response rate from participants, a finding that was consistent with our study [53, 54]. Additionally, attaching the sensor to the participant’s thigh took about two minutes. These findings indicate that participation in this study was not burdensome, which was confirmed by the interviews that showed that 95% of participants felt that participating in the study (i.e., filling out all questionnaires and wearing the ActivPal™ device for 7 days) was not a burden. Overall, ProPASS methods appear to be less burdensome, well-suited, and readily accepted by participants.

The third aspect of feasibility for the baseline ProPASS data collection methodology was the availability of resources and the ability to manage and execute the study. As we aim to create a new cohort adhering to global (ProPASS) standards, protocol training was vital to obtain quality outcomes as per the ProPASS protocol. As a result, the protocol training took around four hours which was similar to a prior study [45]. In terms of the availability of resources, all essential equipment was always accessible. The study also considered skin irritation as an important factor. One study noted that 38% of participants stopped using ActivPal™ due to skin irritation from PALstickies or Tegaderm dressings [55]; another reported one discontinuation due to irritation associated with a Tegaderm dressing [56]. In the current study, there were three reported irritations, with two having mild initial discomfort that eventually subsided. One participant left the study due to moderate irritation. Nonetheless, it is important to note that the data collection occurred during colder winter periods (average 20 degrees Celsius). It is possible that instances of skin irritation could be more pronounced during Saudi Arabia’s hot summer season, characterized by temperatures of approximately 40 degrees Celsius. Future studies should investigate the feasibility of using devices and tape suitable for summer temperatures. In addition, the current study also had a low accelerometer failure rate: only four accelerometers failed to record, which is similar to previous studies [57, 58]. All ActivPal™ devices were returned at the end of the study during visit two, ensuring that the ProPASS method is suitable to be used in future cohorts in Saudi Arabia.

### Strengths and limitations of Study

This study represents the first of its kind to utilize device-based measures for assessing physical behaviors among adults in Saudi Arabia. The device-based measure has been shown to provide useful information about physical behaviors when compared to using self-report questionnaires [16]. Furthermore, it marks the initial examination of the ProPASS consortium method in the Middle East, particularly in Saudi Arabia. Nevertheless, the current study has certain limitations including recruiting among relatively young participants, presumably without any medical conditions and with postgraduate qualifications. This may limit the generalization of the findings to the entire population. The acceptability of the study in other age groups and among individuals with lower educational backgrounds is yet to be studied. In addition, the feasibility of the baseline ProPASS data collection methodology study was conducted during winter, which might have influenced the observed levels of physical behaviors in our sample. Similarly, the study was unable to evaluate the feasibility of utilizing 3 M Tegaderm dressings in hot summer months. Lastly, it’s important to note that our study employed a relatively small sample size; nonetheless, this size is considered acceptable for feasibility studies.

## Conclusion

The baseline ProPASS data collection methodology and protocol for a future cohort study are both feasible and acceptable for implementation within the context of Saudi Arabia. This feasibility study represents the first step toward establishing a prospective ProPASS cohort study to examine the association between physical behaviors and cardiometabolic health among Saudi Arabian adults.

### Supplementary Information

Supplementary Material 1.

Supplementary Material 2.

Supplementary Material 3.

## Data Availability

The datasets used and/or analyzed during the current study available from the corresponding author on reasonable request.

## Appendix

**Table:**

**Feasibility construct**	**Primary**	**Follow-up**
**Motivation, expectations**	**1a.** What made you want to join this study? *If the participant decided to not take part in the study the follow question will be asked* **1.1a. **What factors influenced your decision to not participate in the study?	**1b. **what are some of the most helpful things about the study?
**Satisfaction**	**2a.** What was your overall experience of participating in the study?	**2b.** Was it as you expected?
**Burden**	**3a.** Did you have any burden being participating in the questionnaires? **4a.** Did you have any burden being participating in the physical exams? **5a.** Did have any burden during the week you wore the sensor? **6a.** Were there any moments when you wanted to stop?	**6b. If “Yes”: Why?**
**Willingness to participate again**	**7a. **Would you be willing to participate again, if the measurements were carried our once a year?	**7b.** If “No”: Why?
**Perception of time use**	**8a.** How did you feel about the time it took to participate in this project over seven days? **9.a** Can you tell me how you feel about the project, when you consider how much time you used and how much you feel you gained from participating	

## Abbreviations

ProPASS: The Prospective Physical Activity, Sitting and Sleep consortium
Physical behaviors: Physical activity, sedentary behavior, and sleep
