# Supplementary material for: Feasibility and acceptability of a cohort study baseline data collection of device-measured physical behaviors and cardiometabolic health in Saudi Arabia: expanding the Prospective Physical Activity, Sitting and Sleep consortium (ProPASS) in the Middle East
Source: BMC Public Health. 2024 May 22;24:1379. doi: 10.1186/s12889-024-18867-2 (PMC11112840; doi:10.1186/s12889-024-18867-2)

[redacted]

6 valid days (7 days recording)

	Mo	Tu	We	Th	Fr	Sa	Su
8th May							
15th May							
22nd May							

valid invalid current page

[redacted] (Days 1 to 7)

Fri
12 May 2023

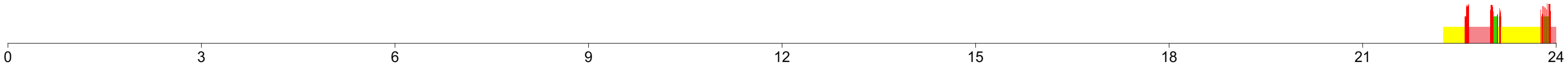

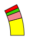

Steps
894
Sit to Stands
4
Activity Score
2.57 MET.h

0h 0.2h
0h 0.2h
1h 0h
0.4h

Sat
13 May 2023

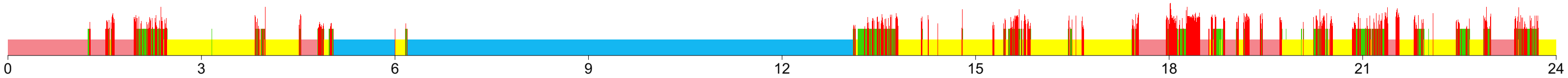

Steps
8728
Sit to Stands
66
Activity Score
33.89 MET.h

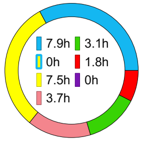

Sun
14 May 2023

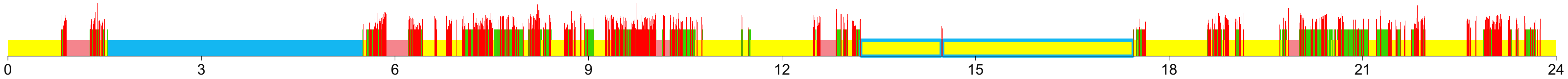

Steps
10922
Sit to Stands
77
Activity Score
34.99 MET.h

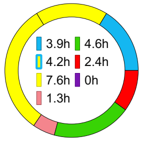

Mon
15 May 2023

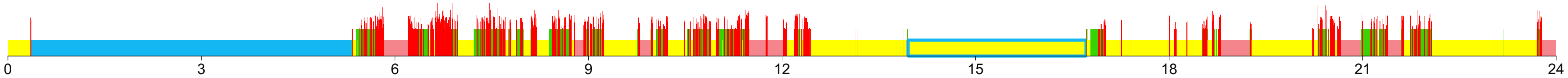

Steps
9768
Sit to Stands
64
Activity Score
34.38 MET.h

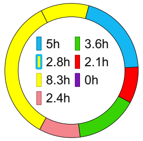

Tue
16 May 2023

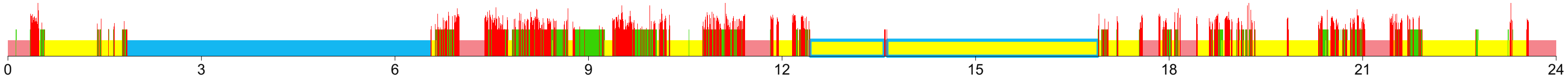

Steps
10060
Sit to Stands
69
Activity Score
34.58 MET.h

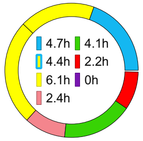

Wed
17 May 2023

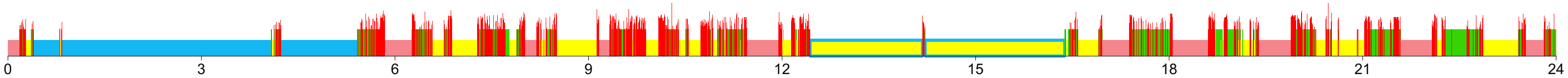

Steps
12140
Sit to Stands
60
Activity Score
35.49 MET.h

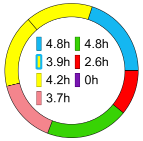

Thu
18 May 2023

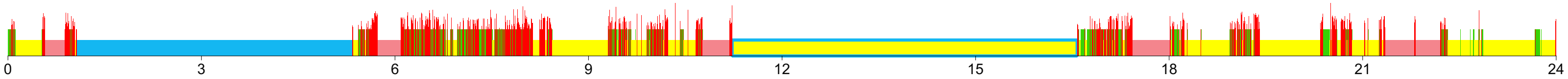

Steps
8692
Sit to Stands
74
Activity Score
33.97 MET.h

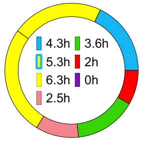

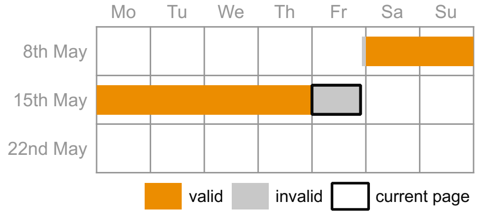

[redacted] (Days 8 to 8)

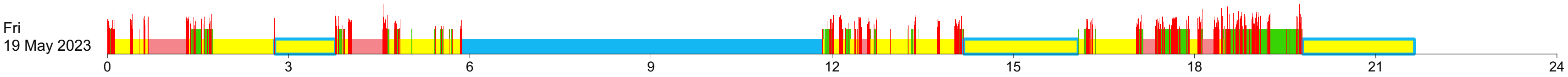

Steps
5848
Sit to Stands
61
Activity Score
29.79 MET.h

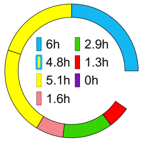

Supplement: Supplementary file 1 — Supplementary Material 1. [file 12889_2024_18867_MOESM1_ESM.pdf]
