# Supplementary material for: Feasibility and acceptability of a cohort study baseline data collection of device-measured physical behaviors and cardiometabolic health in Saudi Arabia: expanding the Prospective Physical Activity, Sitting and Sleep consortium (ProPASS) in the Middle East
Source: BMC Public Health. 2024 May 22;24:1379. doi: 10.1186/s12889-024-18867-2 (PMC11112840; doi:10.1186/s12889-024-18867-2)
Supplement: Supplementary file 2 — Supplementary Material 2. [file 12889_2024_18867_MOESM2_ESM.docx]

| Variables |  | Burden | |  |
| --- | --- | --- | --- | --- |
|  |  | Yes, n (%) | No, n (%) | P* |
| **Sex** | Female | 2 (100) | 18 (46.2) | 0.32 |
|  | Male | 0 (0) | 21 (53.8) |  |
| **Marital status** | Single | 1(50) | 14(35.9) | 0.7 |
|  | Married | 1(50) | 23(59) |  |
|  | divorce | 0(0) | 2(5.1) |  |
| **Education** | High school | 0 (0) | 4(10.3) | 0.62 |
|  | undergraduate | 2(100) | 21(53.8) |  |
|  | post-graduate | 0 (0) | 14(35.9) |  |
| **Employment Status** | Employed | 2(100) | 36(92.3) | 0.58 |
|  | Unemployed | 0 (0) | 1 (2.6) |  |
|  | Homemaker | 0 (0) | 0 (0) |  |
|  | Unpaid voluntary work | 0 (0) | 1 (2.6) |  |
|  | Retired | 0 (0) | 1 (2.6) |  |
| **Willing to participate in the future** | Yes | 2(100) | 37(94.9) | 0.61 |
|  | No | 0(0) | 2(5.1) |  |

Table 3 Compare participants who reported burden of participation with no burden of participation using Chi-Square.

*Chi-square test of difference between groups. Results with P < .05 are indicated in bold.
