# Supplementary material for: Feasibility and acceptability of a cohort study baseline data collection of device-measured physical behaviors and cardiometabolic health in Saudi Arabia: expanding the Prospective Physical Activity, Sitting and Sleep consortium (ProPASS) in the Middle East
Source: BMC Public Health. 2024 May 22;24:1379. doi: 10.1186/s12889-024-18867-2 (PMC11112840; doi:10.1186/s12889-024-18867-2)
Supplement: Supplementary file 3 — Supplementary Material 3. [file 12889_2024_18867_MOESM3_ESM.docx]

Table 4 Comparing continuous variables between participants who reported a burden with no burden of participation.

| Variables | Burden | |  | | | |
| --- | --- | --- | --- | --- | --- | --- |
|  | Yes | No | t | MD [95% CI] | p | d |
| **Age (years)** | 35.5 | 39.13 | 0.694 | 3.63 [-6.94; 14.20] | 0.56 | 0.50 |
| **BMI (kg/m^2^)** | 24.66 | 28.4 | 0.894 | 3.74 [-4.71; 12.20] | 0.45 | 0.65 |
| **SED (min/day)** | 477.00 | 476.00 | -0.008 | −0.69 [-74.92; 173.54] | 0.38 | -0.01 |
| **LPA (min/day)** | 67.70 | 62.75 | -0.29 | −4.94 [-39.30; 29.40] | 0.21 | -0.21 |
| **MVPA (min/day)** | 61.00 | 58.31 | -0.18 | −2.70 [−32.86; −27.48] | 0.28 | -0.13 |
| **Sleep(min/day)** | 331.23 | 400.85 | 0.71 | 69.61 [-129.25; 268.48] | 0.39 | 0.52 |

Abbreviation: BMI, Body Mass Index; SED, sedentary behavior; LPA, Low Physical activity; MVPA, moderate-to-vigorous physical activity.
